# Phosphoinositide 3-kinase δ activity in patients with systemic lupus erythematosus

**DOI:** 10.3389/fimmu.2026.1745692

**Published:** 2026-02-17

**Authors:** Elham Sadat Mirfazeli, Shalmalee Kharkar, Michel W. P. Tsang-A-Sjoe, Olivier Papapietro, Izabella Niewczas, Agner R. Parra Sanchez, Anita Chandra, Klaus Okkenhaug, Irene E. M. Bultink, Reina E. Mebius, Jonathan Clark, Alexandre E. Voskuyl, Sergey Nejentsev

**Affiliations:** 1 Molecular Cell Biology and Immunology, Amsterdam UMC Location Vrije Universiteit Amsterdam, Amsterdam, Netherlands; 2 Amsterdam Institute for Immunology and Infectious Diseases, Amsterdam, Netherlands; 3 Department of Rheumatology and Clinical Immunology Amsterdam UMC, Amsterdam, Netherlands; 4 Department of Medicine, University of Cambridge, Cambridge, United Kingdom; 5 Babraham Institute, Cambridge, United Kingdom; 6 Department of Clinical Immunology, Cambridge University Hospitals NHS Foundation Trust, Cambridge, United Kingdom; 7 Department of Pathology, University of Cambridge, Cambridge, United Kingdom

**Keywords:** APDS, autoimmunity, biomarker, PI3Kδ, PIP3, SLE, T cells

## Abstract

**Introduction:**

New biomarkers are needed for better stratification and personalized treatment of Systemic Lupus Erythematosus (SLE). Phosphoinositide 3-kinase δ (PI3Kδ) has been implicated in SLE pathogenesis. Here, we investigated whether a subset of SLE patients has increased PI3Kδ activity after T cell activation.

**Methods:**

T cells were isolated from frozen PBMCs of 108 SLE patients, 19 healthy controls, and one patient with Activated PI3K Delta syndrome (APDS), which provided a benchmark of increased PI3Kδ activity. After 90-minute anti-CD3/CD28 stimulation, phosphatidylinositol 3,4,5-trisphosphate (PIP3) and phosphatidylinositol 4,5-bisphosphate (PIP2) were measured using high-performance liquid chromatography-mass spectrometry.

**Results:**

Higher levels of PIP3 (measured as the ratio of PIP3/PIP2) in stimulated T cells distinguished APDS patient from other subjects providing a useful biomarker of increased PI3Kδ activity. We observed no significant difference in T-cell PIP3 levels between SLE patients and healthy controls. However, a subset of SLE patients (n = 4) exhibited strong upregulation of PIP3 following T-cell stimulation, comparable to that observed in the APDS patient. PIP3 levels in stimulated T cells positively correlated with the frequency of CD4+ T cells and negatively correlated with the frequencies of CD8+, EMRA CD4+, and EMRA CD8+ T cells.

**Conclusions:**

We describe the range of variation of PI3Kδ activity in T cells from a large cohort of patients with SLE and from healthy subjects. Our findings suggest that increased PI3Kδ activity is not associated with SLE in general, although some SLE patients exhibit a particularly strong upregulation of PIP3 levels after T-cell stimulation. This subgroup of SLE patients warrants further investigation given the promising effect of PI3Kδ inhibitors in restoring normal immune regulation.

## Introduction

Systemic Lupus Erythematosus (SLE) is an autoimmune disease affecting over 3 million people globally. SLE significantly impacts patients’ life through chronic pain, fatigue, disability and dysfunction of vital organs imposing substantial physical, mental and socio-economic challenges on patients. SLE predominantly affects women, with over 90% of cases occurring in females, particularly during the child-bearing years. Disease etiology is complex resulting from interplay between environmental factors and genetic predispositions that trigger an aberrant autoimmune response (1). The clinical manifestations and severity of SLE are highly heterogeneous, complicating both treatment choices and the conduct of clinical trials. Therefore, there is a need to identify biomarkers that can aid in stratifying and managing SLE patients more effectively.

Phosphoinositide 3-kinases (PI3Ks) are lipid kinases that phosphorylate phosphoinositides at the 3-OH position. PI3Ks are grouped into three classes (I, II, and III) based on the subunit structure, substrate specificity, and final phosphoinositide products. PI3Kδ, a class I enzyme predominantly found in leukocytes, is activated by upstream receptor tyrosine kinases (RTKs), T-cell receptor (TCR), B-cell receptor (BCR), and cytokine receptors. PI3Kδ is the main signal transducer of PI3K signaling downstream of TCR in human T cells (2). PI3Kδ is a heterodimeric enzyme consisting of the catalytic subunit p110δ, which most commonly assembles with the regulatory subunit p85α. Upon activation, PI3Kδ converts phosphatidylinositol 4,5-bisphosphate (PIP2) to phosphatidylinositol 3,4,5-trisphosphate (PIP3), an important second messenger that activates signaling pathways through AKT, TEC family kinases, and other PH domain proteins. These pathways in turn largely impact lymphocyte development, proliferation, metabolism, migration, and survival (3).

Enhanced PI3Kδ activity may contribute to the pathogenesis of SLE, as inhibition of the enzyme limits disease progression and improves survival in lupus mouse models. Heterozygous inactivation of the p110δ gene exerts an inhibitory effect on immune compartments, reducing serum IgG anti-nuclear antibodies (ANA) levels, dampening T-cell activity and attenuating glomerulonephritis (4). Pharmacologic inhibition of p110δ also decreased the production of proinflammatory cytokines and several lymphocyte populations, such as Th17 cells, CD3+CD4−CD8−B220+ cells, CD4+ effector memory T cells, as well as activated GL-7+IgG- germinal center (GC) B cells, IgG+ class-switched B cells, and plasma cells (5, 6). It also reduces *de novo* and memory recall responses in lupus mouse models (7).

Overactivation of PI3Kδ can lead to autoimmune manifestations and lupus-like phenotypes in humans and mice. A well-studied example of this is Activated PI3K Delta Syndrome (APDS), which is an inborn error of immunity caused by activating mutations in the genes *PIK3CD* and *PIK3R1* encoding for PI3Kδ subunits p110δ and p85α respectively (8) (9). APDS patients suffer from immunodeficiency and recurrent upper respiratory tract infections, but also 34% of patients showed signs of autoimmunity or inflammatory disease (10, 11). The autoimmune features can resemble that of SLE patients to the level that at least 4 patients fulfilling ACR classification criteria for SLE turned out to have activating mutations in the *PIK3CD* gene (11–13), which demonstrates that APDS can manifest with an SLE-like phenotype. Additionally, elevated levels of anti-nuclear antibodies (ANA) have been observed in the sera of mice with the activating mutation in the *Pik3cd* gene (14). PI3Kδ inhibition was shown to be effective in patients with APDS. Leniolisib, an oral small-molecule selective inhibitor of the PI3Kδ enzyme, has successfully improved immune dysregulation and immunodeficiency in APDS patients and was recently approved for treating APDS (15, 16). Given the evidence for the elevated PI3Kδ activity in SLE pathogenesis, PI3Kδ inhibitors may also be beneficial in treating patients with SLE. However, owing to the high heterogeneity of the disease, this effect may not be observed in all patients, highlighting the need to identify a subgroup of SLE patients with elevated PI3Kδ activity.

The activity of the PI3Kδ enzyme can be determined *ex vivo* in leukocytes by measuring PIP3 levels using high-performance liquid chromatography-mass spectrometry (HPLC-MS) or by measuring intracellular mediators of PIP3 signaling, such as phosphorylated proteins AKT (p-AKT) or S6 (p-S6), using western blotting or flow cytometry. The advantage of the HPLC-MS assay is that it provides a direct and quantitative measure of PIP3, the product of the PI3Kδ enzyme (17).

Here, we assessed the activity of the PI3Kδ enzyme by studying PIP3 levels using HPLC-MS in T cells isolated from a cohort of patients with SLE and healthy individuals. We also investigated the relationship between levels of PIP3 in T cells and the frequency of different T-lymphocyte subpopulations present in the blood of these subjects. We found no difference in the T-cell PIP3 levels between SLE patients and healthy controls. However, some of the SLE patients show particularly strong increase of PIP3 in response to T-cell stimulation comparable to that of the APDS patient.

## Patients and methods

### Human samples and data

All SLE patients included in this study were diagnosed clinically and fulfilled the 1997 American College of Rheumatology revised criteria for the classification of SLE (18). Patients were treated at Amsterdam UMC hospitals and were 18 years of age or older upon the day of inclusion. The data collected from SLE patients included cumulative clinical disease manifestations, laboratory investigations (presence of anti-double-stranded DNA antibodies, anti-Smith antibodies, and anti-phospholipid antibodies), disease state, and current medication use. Due to the ongoing COVID-19 pandemic at the time of sample collection (between June 2020 and August 2021), physical assessment of the SLE patients and, therefore, measuring the disease activity was not feasible. Alternatively, the disease state was categorized as either stable or unstable. The stable disease state was defined as an acceptable level of disease activity that did not require changes in medication and was not preceded by a recent flare. Otherwise, SLE patients were classified as unstable. Controls were healthy adult volunteers. Demographic data collected from all study participants included gender, age, and ethnicity. Additionally, a blood sample was collected from an adult patient with the E81K mutation in the *PIK3CD* gene previously diagnosed with APDS. The study was approved by the VUmc Medical Ethics Committee (2020.169 (A2020.256)) and UK Local Research Ethics Committee (15/WS/0019). All participants signed informed consent for participating in the study.

### T cell stimulation assay

PBMCs were isolated from blood samples using SepMate™ tubes (STEMCELL Technologies, Cat. No. 85450) according to the manufacturer’s protocol. Isolated PBMCs were resuspended in 10% DMSO/FBS solution and stored in liquid nitrogen for long-term preservation. On the day of the experiment, PBMC samples were thawed in a water bath and added dropwise to 10% FBS/RPMI media. Defrosted PBMC samples were depleted of non-T cells using the Pan T Cell Isolation Kit (Miltenyi Biotec, Cat. No. 130-096-535). Briefly, PBMC samples were labeled with a cocktail of biotin-conjugated monoclonal antibodies against CD14, CD15, CD16, CD19, CD34, CD36, CD56, CD123, and CD235a (Glycophorin A), after which they were labelled with magnetic anti-biotin MicroBeads. The cell suspensions were then transferred to LS columns (Cat. No. 130-042-401, Miltenyi Biotec) in the magnetic field of a MACS separator (Miltenyi Biotec), and the non-labeled T cells were washed through and collected.

Isolated T cells were counted, and around 600,000 T cells were transferred to 2 ml Eppendorf tubes per condition to a final volume of 170 µl. Samples were incubated at 37 °C for 1 hour. Thereafter, samples either remained unstimulated or were stimulated using Dynabeads Human T-Activator CD3/CD28 beads (Thermo Fisher, Cat. No. 11131D) using a 1:1 cell-to-bead ratio. All samples were snap-frozen and stored in -70 °C freezers at the end of the stimulation period for HPLC-MS.

### Flow cytometry

The flow cytometry analysis of PBMCs of SLE patients and healthy controls was performed previously (Mirfazeli et al., submitted). Here we analyzed the following lymphocyte subsets: CD4+ and CD8+ T cells, CD3+CD45RA+CD27– effector memory re-expressing CD45RA (EMRA) CD4+ and CD8+ T cells, CD3 +CD27+CD45RA+ naïve CD4+ and CD8+ T cells, and CD3 +CD4+CXCR5+CD45RA– circulating T follicular helper (cTfh) cells, CD19+IgD+CD27-CD38++CD24++ transitional B cells (TrB), CD19+IgD+CD27- naïve B cells, CD19+CD27-IgD- double-negative (DN) B cells and CD19+IgD-CD27+CD38++ plasmablasts.

### PIP3 and PIP2 quantification

Phosphoinositides were measured using high-performance liquid chromatography-mass spectrometry (HPLC-MS) (17). In brief, the samples were extracted using a modified Bligh and Dyer extraction followed by derivatization with TMS-diazomethane. The samples were then analyzed on a ABSciex QTRAP 4000 mass spectrometer as described previously (17).

### Statistical analysis

All the statistical analyses and data plotting were done in GraphPad Prism v10.2.0 (GraphPad Software, Boston, Massachusetts, USA). Outlier identification was performed using the ROUT method (19) with an FDR (q) value set to 5%. Statistical significance was determined using Mann-Whitney U test. The correlation between normalized values of PIP3/PIP2 and the frequency of lymphocyte subpopulations was assessed using the non-parametric Spearman correlation test, and two-tailed P-values were reported. A simple linear regression model was used for curve fitting. P-values were corrected using Bonferroni correction.

## Results

### Patient and healthy donor characteristics

Blood samples were collected from 108 patients with SLE and 19 healthy individuals. Of the SLE patients, 90% were female, compared to 53% of the healthy controls, highlighting the well-documented skewness of SLE toward females in the population (**Table 1**). The median age of the SLE patients was 49 years (interquartile range [IQR]: 38-56), while the median age of the healthy controls was 33 years (IQR: 28-43). Most individuals in both cohorts were of Caucasian descent. Detailed characteristics of the patients and healthy controls as well as the treatment regimen are provided in **Table 1**. Additionally, we studied a blood sample from a 55-year-old male APDS patient. The patient did not receive PI3Kδ inhibitors prior to the blood sample collection.

**Table 1:** Demographic and clinical characteristics of SLE patients and healthy controls.

	Patients with SLE (n=108)	Healthy controls (n=19)
Demographic features		
Gender		
Gender		
Female	98 (91%)	10 (53%)
Male	10 (9%)	9 (47%)
Age (years)	49 (38-56)	33 (28-43)
Ethnicity		
Caucasian	81	18
other	27	1
Disease characteristics		
Disease state		
Stable	85/99 (86%)	**–**
Unstable	14/99 (14%)	**–**
SLE manifestations		–
Arthritis	48/61 (79%)	–
Serositis	19/61 (31%)	–
Nephrological manifestations	28/59 (47%)	–
Neurological manifestations	3/61 (5%)	–
Haematological manifestations	56/61 (92%)	–
Cutaneous manifestations	60/61 (98%)	–
Anti-dsDNA antibody	52/61 (85%)	–
Anti-Sm antibody	12/61 (20%)	–
Antiphospholipid antibodies (aPL)	15/61 (25%)	–
Current medication n=104		
No medication	6 (6%)	–
Hydroxychloroquine (HCQ)	81 (78%)	–
Prednisone	33 (32%)	–
Methotrexate	2 (2%)	–
Mycophenolate mofetil	15 (14%)	–
Sulfasalazine	8 (8%)	–
Azathioprine	19 (18%)	–
Leflunomide	3 (3%)	–
Anti B cell monoclonal antibody therapy (rituximab or belimumab)	2 + 8 (10%)	–
No data	2 (2%)	–

Data are n (%) or median (interquartile range). Systemic lupus erythematosus (SLE) manifestations and treatments are shown as cumulative data based on the most recent information available. The disease state (stable or unstable) was determined by changes in medications and the incidence of flare-ups during clinical visits before and after blood collection. Note that some clinical data were not available for all patients, with n shown in the denominator in the fractions.

### Optimizing the PI3Kδ stimulation assay

**Figure 1:**
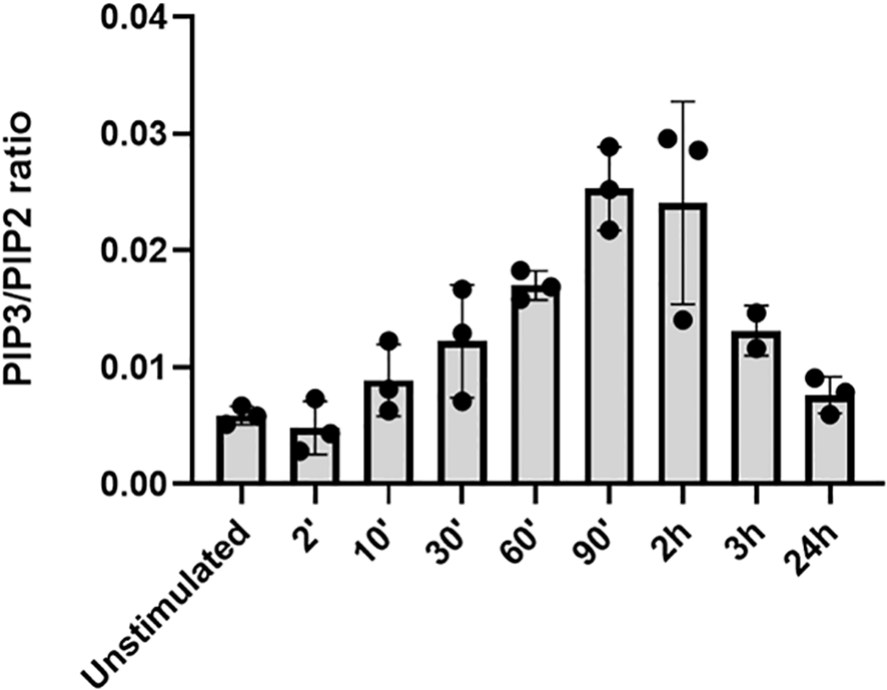
PI3Kδ activation in T cells at different stimulation time-points. T cells were left unstimulated or were stimulated for 2 min, 10 min, 30 min, 60 min, 90 min, 2 h, 3 h, and 24 h in three different test samples, and PIP3/PIP2 was measured using HPLC-MS.

To determine the optimal stimulation time for measuring PI3Kδ activity, T cells were isolated from defrosted PBMCs of three different healthy donors (Sanquin Blood bank, Amsterdam, Netherlands) and stimulated for 2 min, 10 min, 30 min, 60 min, 90 min, 2 h, 3 h, and 24 h with anti-CD3/CD28 beads. At the end of each stimulation period, the samples were snap-frozen. PIP3 and PIP2 were measured using HPLC-MS as described previously (17). PIP3 levels were calculated as the ratio of PIP3/PIP2, which corrects for cell numbers and provides a more accurate measurement of PIP3. The results showed that PIP3 levels peaked at around 90 minutes – 2 hours after stimulation and then decreased (**Figure 1**). Therefore, we chose 90 minutes stimulation for the analysis in the samples of the whole cohort.

### Some SLE patients exhibit strong upregulation of PI3Kδ activity

**Figure 2:**
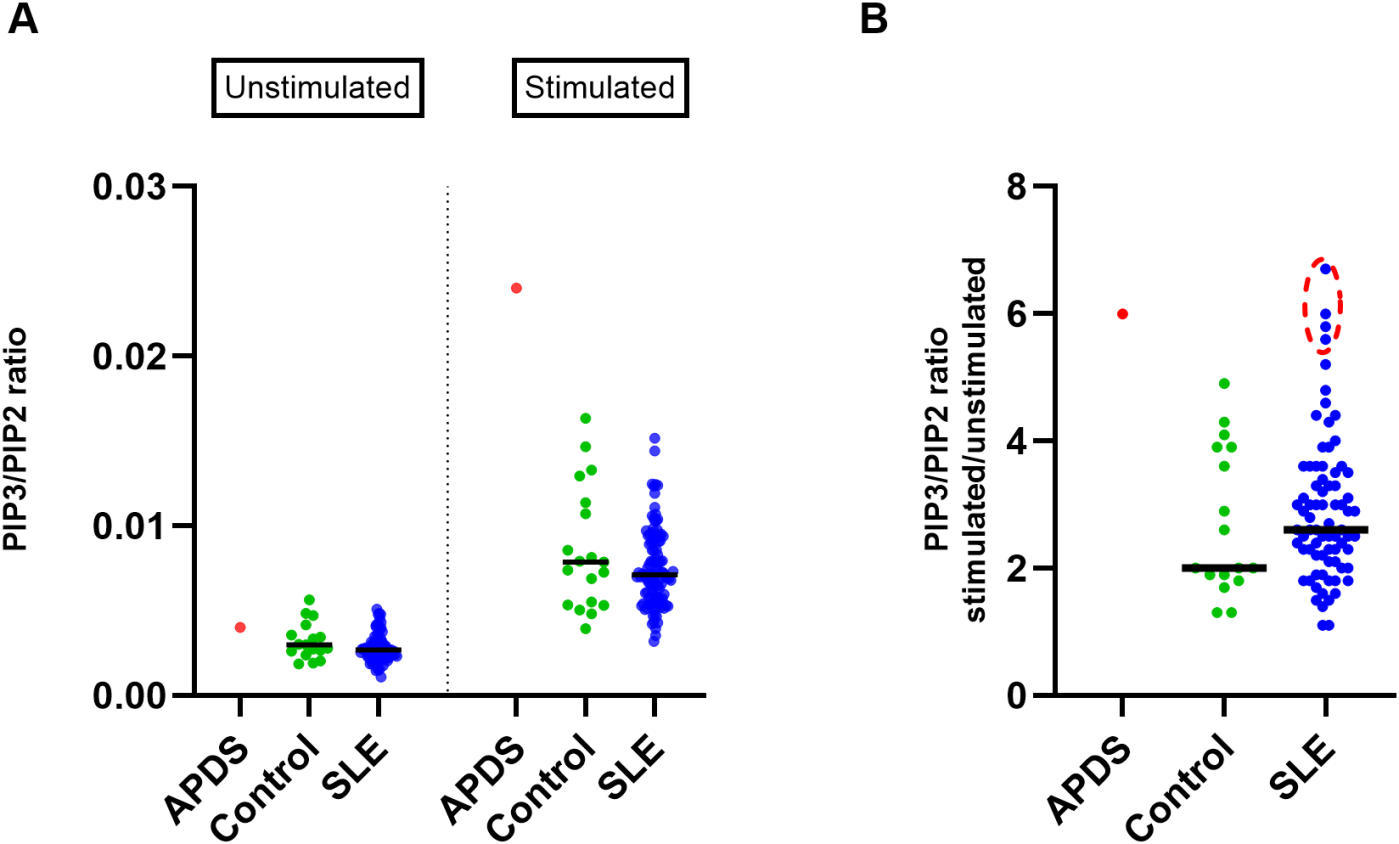
Analysis of PIP3 levels in T cells in the cohorts of SLE patients and healthy controls **(A)** Scatter plot showing PIP3 levels (measured as PIP3/PIP2 ratio using HPLC-MS) in T cells isolated from the blood of SLE patients (n=108), healthy controls (n=19), and an APDS patient before and after 90 minutes of stimulation with anti-CD3/CD28 beads. **(B)** Fold change of PIP3/PIP2 in stimulated over unstimulated T cells; SLE patients (n=77), controls (n=17). Extreme data points in the SLE cohort (circled in red) were calculated using ROUT outlier identification method at FDR = 5%. Median values are indicated by horizontal lines.

To investigate the activity of the PI3Kδ enzyme, we measured PIP3 levels in isolated T cells without stimulation and after 90 minutes of stimulation with anti-CD3/CD28 beads and compared them between SLE patients, healthy controls and the APDS patient. Analysis of unstimulated samples showed only slightly increased PIP3 in the APDS patient and similar levels in SLE patients and controls (**Figure 2a**), indicating that baseline PIP3 levels in unstimulated samples alone are not useful as a biomarker of PI3Kδ activity. Stimulation for 90 minutes led to the increased PIP3 levels in all groups (**Figure 2a**). Analysis of stimulated cells showed that the APDS patient had higher PIP3 levels than SLE patients or healthy controls. We did not find significant difference in PIP3 levels between SLE patients and controls (P = 0.36), and the variation was similar in both groups (**Figure 2a**).

We then studied PIP3 upregulation as the fold change in stimulated over unstimulated cells, a metric that shows how strongly cells respond to stimulation (**Figure 2b**). Again, we found that response to stimulation was strongest in the APDS patient. On average, SLE patients showed somewhat stronger response than controls, but the difference was not significant (P = 0.44).

Nevertheless, four SLE patients exhibited a higher fold-change increase in PIP3 from unstimulated to stimulated T cells (ROUT outlier identification method, FDR = 5%), which was comparable to that observed in the APDS patient (**Figure 2b**). The highest increase among SLE patients was found in a 25-year-old female with unstable disease and a history of nephritis. The other three SLE patients had stable disease and did not exhibit any remarkable features in their clinical data compared to those with average or low ratios. These results suggest that some SLE patients exhibit strong upregulation of PIP3 levels comparable to those observed in APDS patients.

### PIP3 levels in stimulated T cells positively correlate with CD4+ T cell abundance

**Figure 3:**
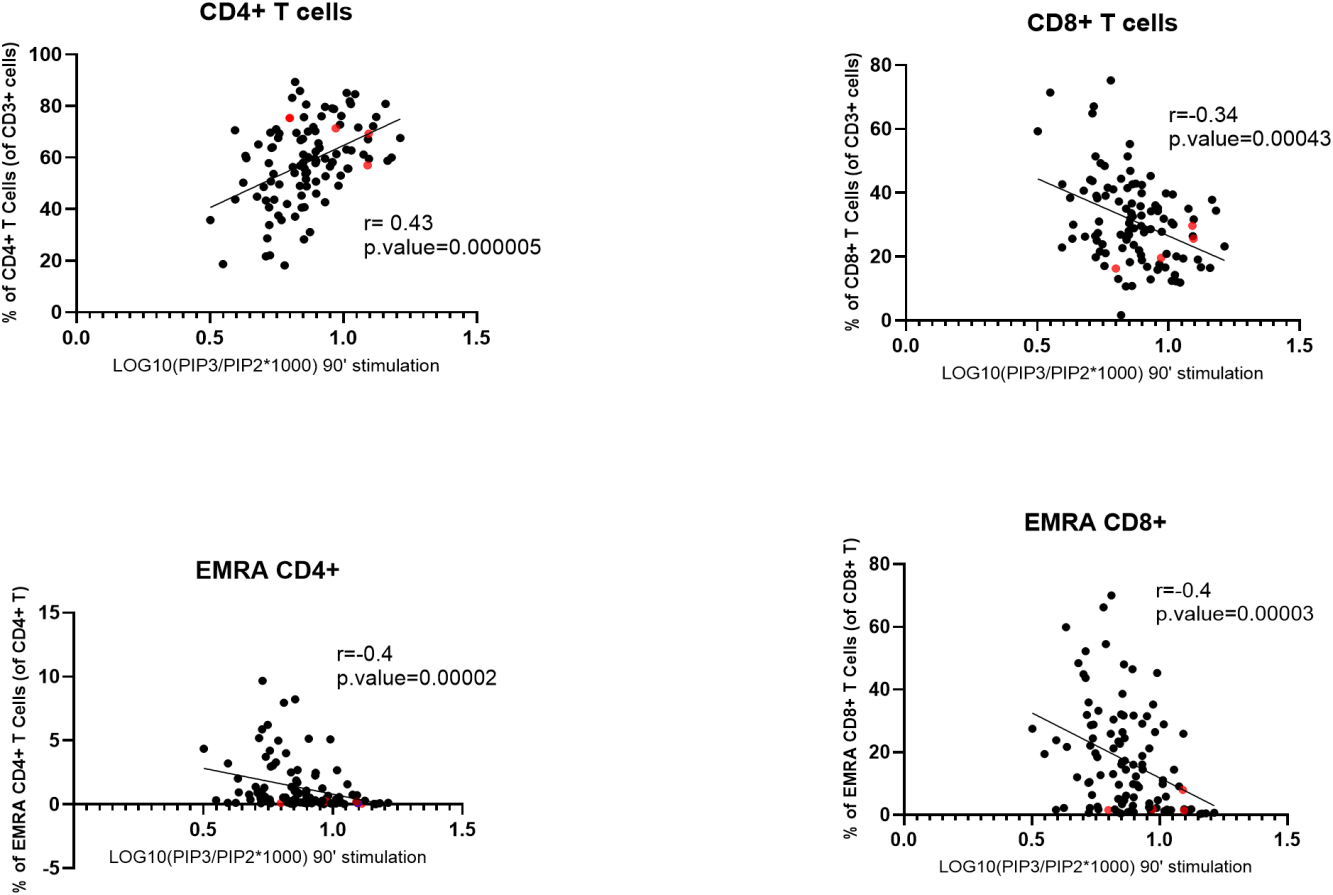
Correlation between normalized PIP3 levels in stimulated T cells and the frequencies of T-cell subpopulations. SLE patients and healthy controls were combined for this analysis. Normalized PIP3 levels were calculated as log10(PIP3/PIP2*1000). Spearman correlation coefficients (r) are shown. P-values were calculated using Mann–Whitney U test. The four SLE patients with extremely high PIP3 fold-change levels are highlighted in red. EMRA, effector memory cells that re-express CD45RA.

To study the role of PI3Kδ in different T-cell subsets, we investigated the relationship between PIP3 levels in stimulated T cells and the frequencies of T-cell subsets found in the blood of the same subjects, which we established previously (Mirfazeli et al., submitted). The results showed a significant positive correlation between normalized PIP3 levels in stimulated T cells and the frequency of CD4+ T cells in the blood (r = 0.43, P_cor_ = 0.00005), while a negative correlation was found for the frequency of CD8+ T cells (r = -0.34, P_cor_ = 0.00043). Similar results were found in SLE patients with both stable and unstable disease (**Supplementary Figure S1**). We also observed negative correlations for the frequencies of CD3+CD45RA+CD27– EMRA CD4+ T cells (r = -0.4, P_cor_ = 0.00002) and EMRA CD8+ T cells (r = -0.4, P_cor_ = 0.00003) (**Figure 3**), while no significant correlation was found for other T cell subsets, such as CD3+CD27+CD45RA+ naïve CD4+ or CD8+ T cells or CD3+CD4+CXCR5+CD45RA– circulating T follicular helper (cTfh) cells (**Supplementary Figure S2**). There was no significant correlation between normalized PIP3 levels measured in stimulated T cells and the frequencies of the B-cell subsets in the blood, including transitional B cells (TrB), naïve B cells, double-negative (DN) B cells or plasmablasts (**Supplementary Figure S2**).

## Discussion

This is the first study to investigate PI3Kδ enzyme activity in a large cohort of SLE patients by measuring PIP3 levels in T cells. Here we used the HPLC-MS assay that allows direct PIP3 quantification (17). The inclusion of a patient with APDS, a disease known for its elevated PI3Kδ activity, provided a benchmark for assessing the increased activity of the enzyme in our study population.

We found that PIP3 levels measured in unstimulated T cells are not a reliable biomarker of increased PI3Kδ activity, as they do not differentiate the APDS patient from healthy controls. However, measuring PIP3 levels in stimulated T cells (after 90 minutes of stimulation) serves as an informative biomarker, which clearly distinguished the APDS patient. Similarly, the fold change of PIP3 levels in stimulated over unstimulated cells is also a suitable biomarker that provided additional information into the cells’ capacity to respond to stimulation and differentiated the APDS patient from healthy subjects.

Our results showed no difference in the PIP3 levels between the groups of SLE patients and healthy controls, suggesting that increased PI3Kδ activity in T cells is not generally associated with SLE. However, several SLE patients upregulated PIP3 levels after T-cell stimulation as strongly as the APDS patient. Previously, elevated PI3Kδ activity was reported in over half of SLE patients studied (20). This was determined using an *in vitro* kinase assay by assessing PIP3 production by PI3Kδ immunoprecipitated from lysed PBMCs, whereas we measured endogenous PIP3 production in T cells. Apart from the assay used, patients with active disease may have contributed to the higher frequency of elevated PI3Kδ activity observed in the study by Suárez-Fueyo et al. compared to our cohort.

In the present study, we found a strong correlation between normalized PIP3 levels in stimulated T cells and the frequencies of T-cell subtypes. In particular, PIP3 levels positively correlated with the frequency of CD4+ T cells and negatively correlated with the frequency of CD8+ T cells. This result is unlikely to be explained by the causative effect of PI3Kδ activity on the T-cell composition of the blood, given that in APDS patients hyperactivated PI3Kδ is known to lead to low CD4+ T cell counts and reduced ratio of CD4+/CD8+ T cells (8) (10, 11). More likely, our results indicate that after anti-CD3/CD28 stimulation CD4+ T cells show higher PI3Kδ activity than CD8+ T cells. This is consistent with the observation that PI3Kδ inhibition has a stronger effect on the proliferation of CD4+ than CD8+ T cells (21). Previous studies have shown that PI3Kδ mediates activation and proliferation of CD4+ T cells (22–24), which may explain its high activity in this cell type after stimulation. While the cytotoxic function of CD8+ T cells is also known to be regulated by PI3Kδ signaling (25, 26), CD8+ T cells become less dependent on PI3Kδ when they are differentiated into memory cells (27). Hence, the negative correlation between PIP3 levels and the frequency of CD8+ T cells in our data is likely to reflect lower PI3Kδ activity in CD8+ in comparison to CD4+ T cells. We also observed a negative correlation between PIP3 levels and the frequency of EMRA CD4+ and CD8+ T cells, suggesting that the presence of such T cells in the blood is associated with reduced PI3Kδ activity. EMRA T cells exhibit characteristics of replicative senescence and reduced proliferative capacity (28), which may be mediated by weakened PI3Kδ activation after stimulation. Overall, such intrinsic differences in PI3Kδ activity suggest that various T-cell subtypes will respond differentially to PI3Kδ inhibitors, which should be taken into consideration in the treatment regimens.

In conclusion, we show the distribution of basal and stimulated PIP3 levels in T cells from healthy subjects and a large cohort of patients with SLE measured for the first time using HPLC-MS and their correlation with T cell subsets. Overall, we found no differences in PI3Kδ activity between the groups of SLE patients and healthy controls. However, we identified individual SLE patients that showed a particularly strong upregulation of PIP3 levels after T-cell stimulation, comparable to that observed in the APDS patient. Given that PI3Kδ inhibitors can reduce responses to T-cell stimulation, this subgroup of SLE patients deserves further studies, as it may benefit from such a treatment.

## Data Availability

The raw data supporting the conclusions of this article will be made available by the authors, without undue reservation.
