# Supplementary material for: Phosphoinositide 3-kinase δ activity in patients with systemic lupus erythematosus
Source: Front Immunol. 2026 Feb 17;17:1745692. doi: 10.3389/fimmu.2026.1745692 (PMC12953358; doi:10.3389/fimmu.2026.1745692)
Supplement: Supplementary file 1 [file DataSheet1.pdf]

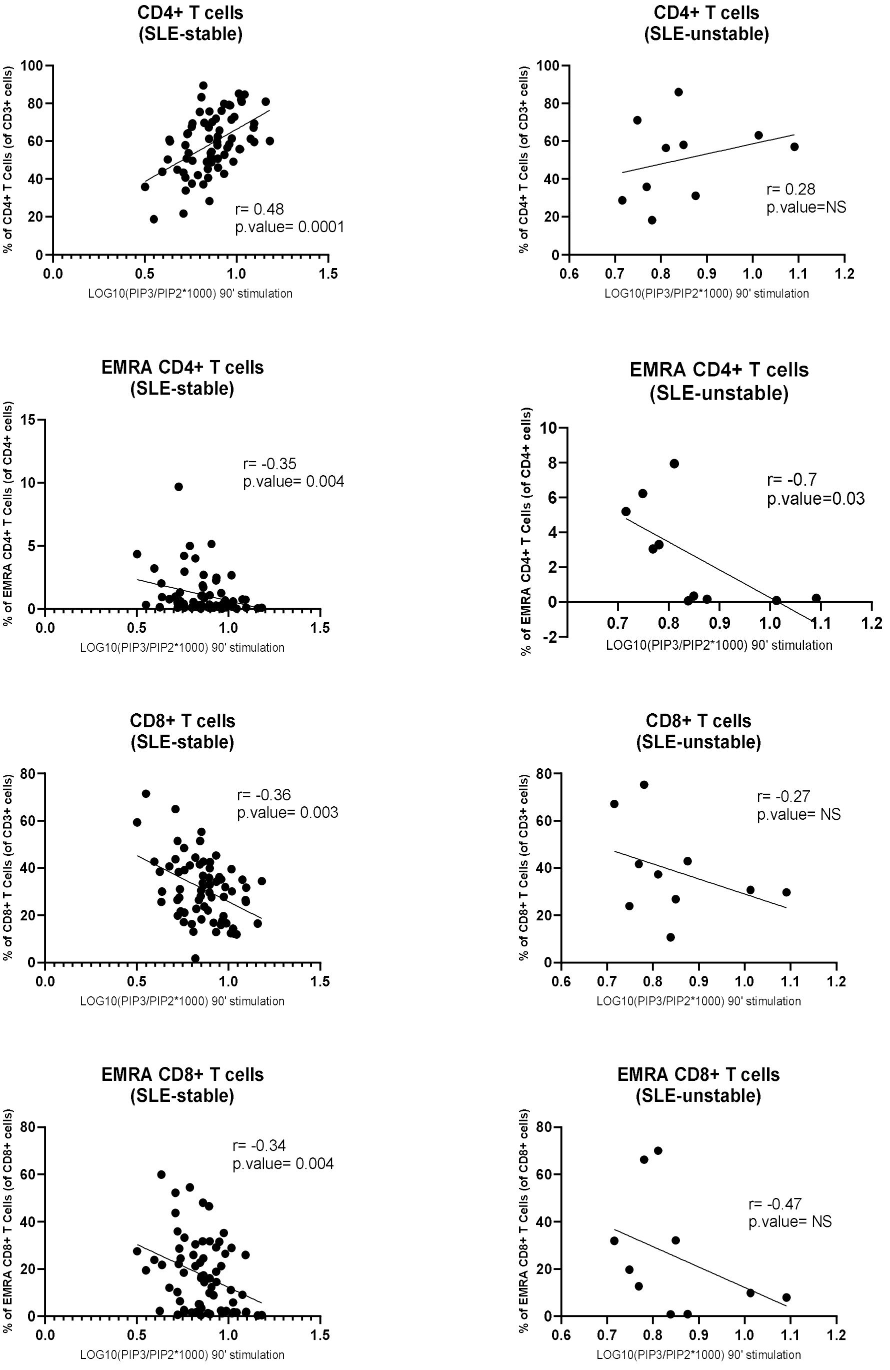

Figure S1. Correlation between normalized PIP3 levels in stimulated T cells and the frequencies of T-cell subpopulations separately in patients with stable or unstable disease.

Normalized PIP3 levels were calculated as log10(PIP3/PIP2*1000). Spearman correlation coefficients (r) are shown. *P*-values were calculated using Mann–Whitney U test. EMRA, effector memory cells that re-express CD45RA.

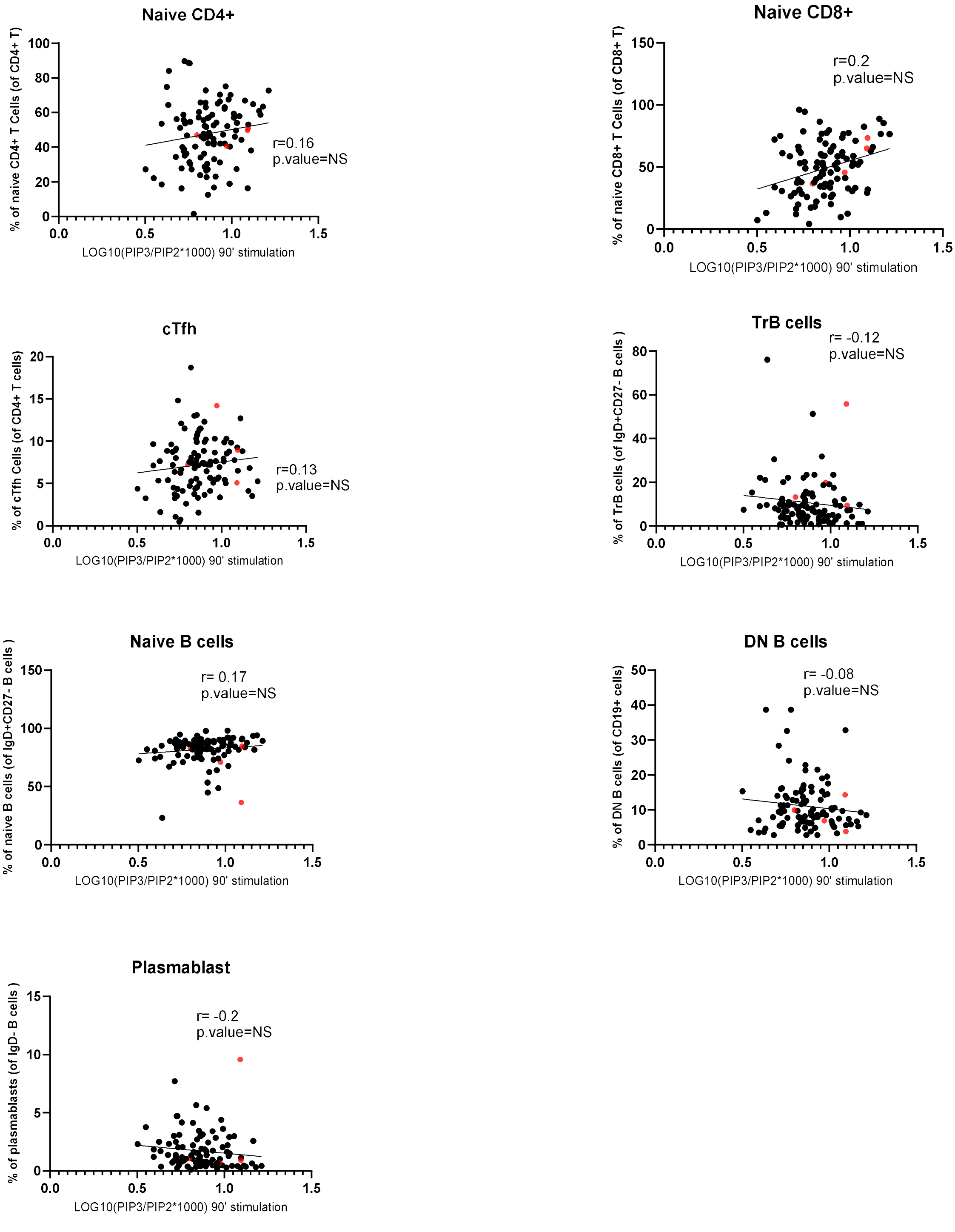

Figure S2. Correlation between normalized PIP3 levels in stimulated T cells and the frequencies of T- and B-cell subpopulations.

SLE patients and healthy controls were combined for this analysis. Normalized PIP3 levels were calculated as log10(PIP3/PIP2*1000). Spearman correlation coefficients (r) are shown. The four SLE patients with extremely high PIP3 fold-change levels are highlighted in red. *P*-values were calculated using Mann–Whitney U test. $P < 0.05$ was considered statistically significant; NS, not significant. cTfh, circulating T follicular helper cells; TrB, transitional B cells; DN, double-negative B cells.
